# Unique case of esophageal rupture after a fall from height

**DOI:** 10.1186/1471-227X-9-24

**Published:** 2009-12-15

**Authors:** Mark van Heijl, Teun P Saltzherr, Mark I van Berge Henegouwen, J Carel Goslings

**Affiliations:** 1 Department of Surgery, Academic Medical Centre, Amsterdam, Netherlands

## Abstract

**Background:**

Traumatic ruptures of the esophagus are relatively rare. This condition is associated with high morbidity and mortality. Most traumatic ruptures occur after motor vehicle accidents.

**Case Presentation:**

We describe a unique case of a 23 year old woman that presented at our trauma resuscitation room after a fall from 8 meters. During physical examination there were no clinical signs of life-threatening injuries. She did however have a massive amount of subcutaneous emphysema of the chest and neck and pneumomediastinum. Flexible laryngoscopy revealed a lesion in the upper esophagus just below the level of the upper esophageal sphincter. Despite preventive administration of intravenous antibiotics and nutrition via a nasogastric tube, the patient developed a cervical abscess, which drained spontaneously. Normal diet was gradually resumed after 2.5 weeks and the patient was discharged in a reasonable condition 3 weeks after the accident.

**Conclusions:**

This case report presents a high cervical esophageal rupture without associated local injuries after a fall from height.

## Background

Esophageal rupture due to external trauma was first described in 1936 by Vinson[1]. Traumatic ruptures of the esophagus represent 4-14% of all esophageal perforations[2,3]. Penetrating or iatrogenic trauma (during endoscopy or operation) are the most frequent causes of rupture of the esophagus[4]. Ruptures of the esophagus due to blunt trauma are very rare and are estimated to occur in less than 1% of patients experiencing blunt (cervical) trauma[4]. We present a rare case of cervical esophageal rupture after a high energy trauma due to a fall from the third floor of a building.

## Case presentation

**Figure 1:**
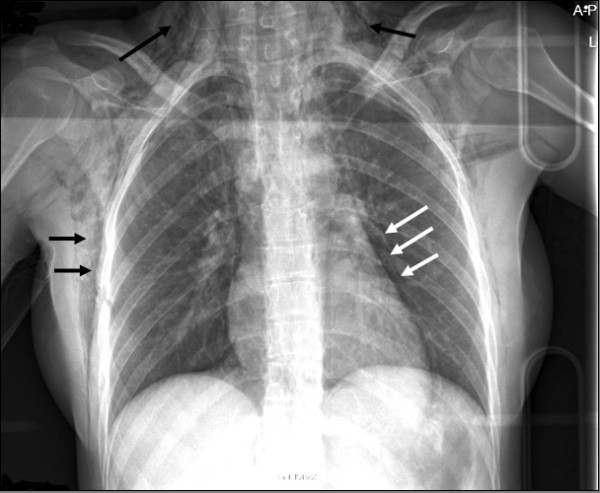
**X-ray of chest on day of injury**. Black arrows: subcutaneous emphysema. White arrows: pneumomediastinum

A 23 year old woman presented at our trauma resuscitation room after a fall from 8 meters. During physical examination (ATLS^® ^protocol) there were no clinical signs of life-threatening injuries. Neurological examination did not reveal any abnormalities either. She did however have a large amount of subcutaneous emphysema of the chest and neck and complained of low back pain. Plain X-rays of the chest confirmed the subcutaneous emphysema of the chest and revealed a pneumomediastinum without signs of pneumothoraces (Figure 1). Due to the massive amount of subcutaneous emphysema the normal X-rays were considered inevaluable and subsequent contrast enhanced Computed Tomography (CT) scanning of the neck and chest was performed. This showed an unstable fracture of the first lumbar vertebra, a fracture of the right inferior pubic ramus, a small right-sided pneumothorax and confirmed the pneumomediastinum. CT also raised suspicion of an esophageal injury, in the absence of large pulmonary or tracheal injuries. After administering oral contrast no contrast leakage could be detected on a second CT scan the same day. Bronchoscopy was also performed and showed no abnormalities. Intravenous antibiotics (Amoxicillin and Metronidazol) were administered for 4 days based on clinical and radiological suspicion of esophageal rupture.

**Figure 2:**
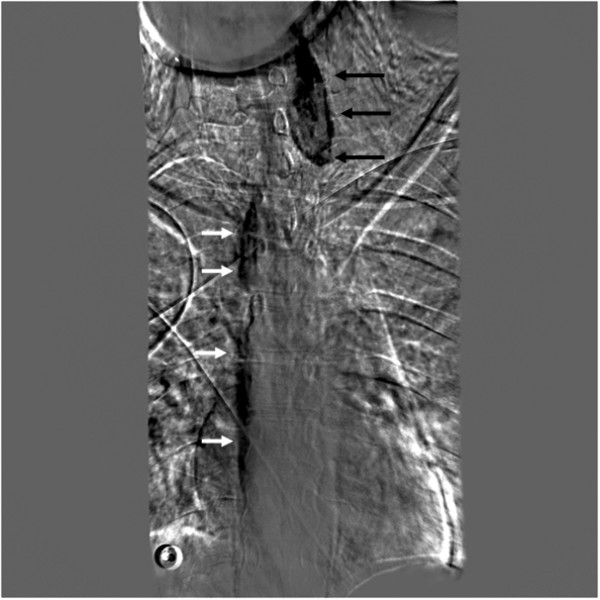
**Contrast swallow examination on day of injury**. Black arrows: contained leakage (contrast extravasation). White arrows: contrast descending in esophagus

An emergency operation was indicated for stabilization of the unstable lumbar vertebral fracture and the patient was observed postoperatively at the Intensive Care Unit (ICU). In this phase the Ear/Nose/Throat specialist (ENT) was consulted and during examination the patient only complained of dysphagia and painful coughing. Subsequently, a flexible laryngoscopy was performed which revealed a lesion in the upper esophagus just under the level of the upper esophageal sphincter. A contrast-swallow examination showed contained leaking of contrast from the posterior wall of the cervical esophagus into the retropharyngeal area next to the esophagus (Figure 2).

**Figure 3:**
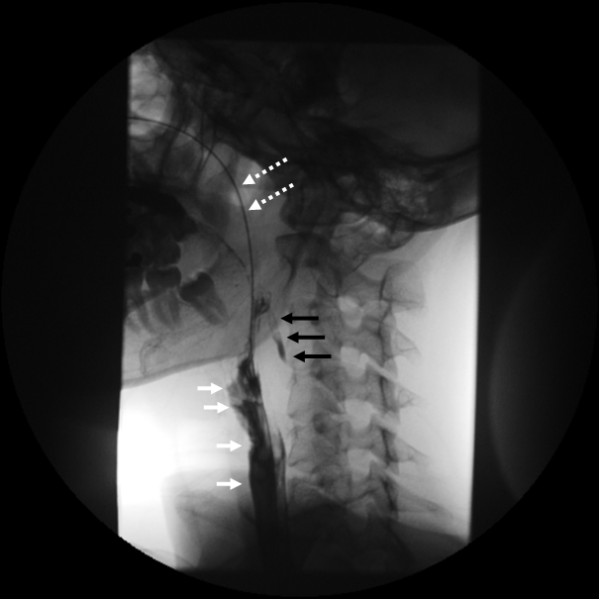
**Contrast swallow video examination on day 10**. Black arrows: contrast leakage (extravasation) in blind sinus. White arrows: contrast continuing in esophagus. White dotted arrows: nasogastric tube.

Based on these findings and the patient's clinically stable condition conservative treatment was initiated consisting of nutrition via a nasogastric tube. A control contrast-swallow video examination on the tenth day after trauma showed minimal contrast extravasation into a blind sinus (Figure 3). An episode of fever and increased infectious laboratory parameters (leukocyte count 15,1 × 10^9^/L and C-reactive protein 17 mg/L) however were reason to restart antibiotics (Amoxicillin) intravenously on the 13^th ^day. The following day the patient also coughed up some purulent fluid and had painful swelling on the left side of the neck, suspicious for an abscess. Laryngoscopy was performed 2.5 weeks after the trauma and showed no abnormalities. X-ray of the cervical spine showed minimal subcutaneous emphysema. Normal diet was gradually resumed and the nasogastric tube was removed. After 3 weeks the patient was discharged. ENT follow-up showed no evidence for continued leakage.

Written informed consent was obtained from this patient.

## Conclusions

Cervical esophageal rupture due to blunt trauma without associated injuries is very rare. Esophageal rupture is associated with high mortality and morbidity; early diagnosis and subsequent treatment can add to a beneficial outcome[2,5]. We present a unique report of a case of a high cervical esophageal rupture after a fall from height without associated injuries in the cervical area. Case reports about traumatic esophageal ruptures are not new; however, almost all cases describe motor vehicle accidents [6-15]. Explanation of the trauma mechanism in case reports on cervical esophageal ruptures specifically vary from blunt external trauma, cervical flexion-hyperextension injury to fracture-dislocation of cervical vertebrae [6,10]. Tracheo-esophageal fistula following a fall of 3 m was reported once, and was surgically repaired[16]. This was however an intrathoracic esophageal rupture located just above the carina and thought to be caused by the esophagus and trachea being crushed between the sternum anteriorly and the vertebral column posteriorly. In our case the trauma-mechanism could not be fully clarified. Because no associated lesions were found in the cervical area, direct blunt trauma is probably not the cause of this rupture. Rupture caused by crush against the cervical spine due to flexion-hyperextension injury has never been described without concomitant cervical spine injury. This leaves an acute rise in intraluminal esophageal pressure as the most probable cause for this rupture.

Another lesson that can be learned from this case is the fact that the leakage was not detected by CT, even after administering oral contrast. Although no specific physical complaints of the injury were present during initial evaluation and the injury itself was not detected on CT high clinical suspicion was raised due to massive subcutaneous emphysema and pneumomediastinum without injury to the trachea, bronchus or lungs on CT and bronchoscopy. This was the main reason to suspect the diagnosis of esophageal rupture, perform laryngoscopy and to start prophylactic antibiotics and conduct further diagnostics, as recommended earlier by Goudarzi *et al *[10]. Contrast-swallow examination and upper esophageal endoscopy are diagnostic modalities of choice in case of suspicion of esophageal rupture[17]. Delay in diagnosis was introduced in our case because other, potentially disabling injuries required treatment first. However, no adverse effects were encountered; antibiotics were already initiated and oral nutrition prohibited.

Depending on the cause and site of a rupture, treatment is either conservative or interventional. Interventional treatment options consist of surgical repair, esophageal resection, exclusion and diversion of the esophagus and chest drainage with or without repair. However, interventional treatment is more frequently required in intrathoracic ruptures. In general, most cervical esophageal perforations unlike intrathoracic perforations can be treated conservatively, especially if the leak is contained and clinical signs are mild[18]. Conservative treatment consists of fluid resuscitation, antibiotics, gastric decompression and food restriction. It is reported that 80% of the conservatively treated high esophageal ruptures will heal successfully[18]. In case of contained leakage it is most unlikely that secondary life-threatening complications like mediastinitis develop, which justifies our policy in this case.

This case report presents a high cervical esophageal rupture without associated local injuries after a fall from height. As with any other cervical esophageal perforations, early recognition and treatment are of great importance. This case supports the selective non-surgical treatment policy for cervical perforations.

## Competing interests

T.P.Saltzherr is a research fellow at the Trauma Unit Department of Surgery, employed by the AMC Medical Research B.V. and supported by an unrestricted grant from Siemens Medical Solutions, Den Haag, the Netherlands.

## Authors' contributions

JCG was the admitting specialist during initial assessment and trauma care of this patient. All four authors drafted, read and approved the final manuscript.

## Pre-publication history

The pre-publication history for this paper can be accessed here:

http://www.biomedcentral.com/1471-227X/9/24/prepub
